# Genetic Diversity and Geographic Distribution of Genetically Distinct Rabies Viruses in the Philippines

**DOI:** 10.1371/journal.pntd.0002144

**Published:** 2013-04-04

**Authors:** Mariko Saito, Hitoshi Oshitani, Jun Ryan C. Orbina, Kentaro Tohma, Alice S. de Guzman, Taro Kamigaki, Catalino S. Demetria, Daria L. Manalo, Akira Noguchi, Satoshi Inoue, Beatriz P. Quiambao

**Affiliations:** 1 Tohoku University Graduate School of Medicine, Sendai City, Miyagi, Japan; 2 RITM-Tohoku Collaborative Research Center on Emerging and Re-emerging Research, Muntinlupa City, Metro Manila, Philippines; 3 Research Institute for Tropical Medicine, Muntinlupa City, Metro Manila, Philippines; 4 National Institute of Infectious Disease, Tokyo, Japan; The Global Alliance for Rabies Control, United States of America

## Abstract

**Background:**

Rabies continues to be a major public health problem in the Philippines, where 200–300 human cases were reported annually between 2001 and 2011. Understanding the phylogeography of rabies viruses is important for establishing a more effective and feasible control strategy.

**Methods:**

We performed a molecular analysis of rabies viruses in the Philippines using rabied animal brain samples. The samples were collected from 11 of 17 regions, which covered three island groups (Luzon, Visayas, and Mindanao). Partial nucleoprotein (N) gene sequencing was performed on 57 samples and complete glycoprotein (G) gene sequencing was performed on 235 samples collected between 2004 and 2010.

**Results:**

The Philippine strains of rabies viruses were included in a distinct phylogenetic cluster, previously named Asian 2b, which appeared to have diverged from the Chinese strain named Asian 2a. The Philippine strains were further divided into three major clades, which were found exclusively in different island groups: clades L, V, and M in Luzon, Visayas, and Mindanao, respectively. Clade L was subdivided into nine subclades (L1–L9) and clade V was subdivided into two subclades (V1 and V2). With a few exceptions, most strains in each subclade were distributed in specific geographic areas. There were also four strains that were divided into two genogroups but were not classified into any of the three major clades, and all four strains were found in the island group of Luzon.

**Conclusion:**

We detected three major clades and two distinct genogroups of rabies viruses in the Philippines. Our data suggest that viruses of each clade and subclade evolved independently in each area without frequent introduction into other areas. An important implication of these data is that geographically targeted dog vaccination using the island group approach may effectively control rabies in the Philippines.

## Author Summary

Rabies continues to be a major public health problem in the Philippines. We conducted a molecular epidemiological study of rabies using the complete glycoprotein (G) gene from 235 animal brain samples collected in the Philippines between 2004 and 2010. We identified three major clades and two distinct genogroups in the Philippines. The three major clades L, V, and M were found specifically in the Luzon, Visayas, and Mindanao island groups, respectively. Additionally, two minor genogroups were located in the Luzon island group. These data suggest that although human mediated transmission may have occurred, these virus clades evolved independently after a single introduction into each island group. All of the analyzed Philippine strains were clustered into Asian 2b, which diverged from the Chinese strain Asian 2a. No recent introduction of rabies into the Philippines from other countries was apparent. The elimination of rabies by 2020 is a national goal in the Philippines, necessitating urgent development of a more effective and feasible strategy for controlling rabies. Our findings indicate that a geographically targeted dog vaccination campaign may effectively control rabies in island nations such as the Philippines.

## Introduction

Rabies is a fatal viral disease that causes an estimated 55,000 human deaths globally ever year, of which 57% occur in Asia [1]. Although effective measures to control rabies, such as dog vaccination, are available, rabies still is a major public health problem in many countries such as the Philippines, where 200–300 human rabies cases were reported annually by the National Notifiable Diseases Surveillance System between 2001 and 2011 [2]. Human rabies cases were also exported from the Philippines to countries that had been declared rabies-free, including Japan in 2006 [3], [4] and Finland in 2007 [5]. The National Rabies Control and Prevention Program of the Philippines is a joint effort by the Department of Agriculture, Department of Health, and other partners that aim to eliminate rabies from the country by the year 2020 [2]. Hence, there is an urgent need to establish an effective and feasible strategy for controlling rabies in the Philippines.

The rabies virus of the *Rhabdoviridae* family is the major Lyssavirus responsible for majority of human and animal rabies cases. The rabies viral genome is a nonsegmented single-stranded negative-sense RNA of approximately 12 kb, which encodes a nucleoprotein (N), a phosphoprotein (P), a matrix protein (M), a glycoprotein (G), and a polymerase (L) [6], [7]. The rabies virus N gene is most commonly used for diagnosis with the reverse transcription-polymerase chain reaction (RT-PCR) because it is the most conserved gene in lyssaviruses [8]. The N gene has also been used extensively in molecular epidemiological studies [7], [9]–[16] and the G gene encodes a surface protein that is targeted by neutralizing antibodies. In addition, this surface protein is crucial to viral invasion of host cells because it attaches to the host receptors [17]. Many recent studies have utilized the rabies G gene for molecular epidemiological analyses [10], [14]–[16], [18], [19].

Phylogenetic analysis of the partial N gene was reported previously for rabies viruses in the Philippines and indicated that these viruses formed a unique phylogenetic group that was further divided into two subgroups [9]. However, only a limited number of specimens were analyzed. Therefore, more detailed genetic information is needed to characterize circulating rabies viruses and to further resolve their transmission dynamics in the Philippines. Understanding the transmission dynamics and genetic diversity of rabies provides useful information for establishing a rabies control strategy [13], [20]. The National Rabies Prevention and Control Program (NRPCP) of the Philippines is implementing a geographically targeted dog vaccination approach to eliminate rabies. As the Philippines is an island nation, this approach should be feasible and effective for eliminating rabies provided transmissions between islands are not frequent. Detailed molecular analyses of rabies viruses from different regions elucidates geographic compartmentalization, evolution, and transmission dynamics, which can be used to establish an effective rabies control strategy in the Philippines.

In the present study, we analyzed the complete G gene (1572 nt) of rabies-positive animal brain samples collected from different regions in the Philippines. We also performed partial N gene sequencing (1124 nt) of the selected samples to compare the results with those of complete G gene sequencing.

## Materials and Methods

The study protocol was approved by the Institutional Review Board of the Research Institute for Tropical Medicine (RITM), which requires a review of all research projects conducted at the RITM.

### Sample Collection

Tissues from the hippocampus and medulla were collected from suspected rabid animals from different regions of the Philippines between 2004 and 2010. These included samples retrieved from the sample bank of the National Rabies Reference Laboratory at the RITM, which were collected from 2004 to 2007. The remaining samples were collected prospectively from 2008 to 2010 from the Regional Animal Disease Diagnostic Laboratories (RADDLs) of Regions 1, 2, 3, 5, 7, and 10, the Cordillera Autonomous Region (CAR) of the Department of Agriculture, and the Provincial Animal Disease Diagnostic Laboratory (PADDL) of Dumaguete City, Negros Oriental. The Philippines is an island nation comprising over 7,000 islands that are divided into three groups, Luzon, Visayas, and Mindanao. The country is governed as 17 regions and 80 provinces. Samples were collected from 11 regions, including Regions 1, 2, 3, 4A, 4B, 5, CAR, the National Capital Region (NCR) in Luzon, Region 7 in Visayas, and Regions 10 and 11 in Mindanao (Figure S1). A fluorescent antibody test (FAT) was performed at the RADDLs and PADDL to diagnose rabies infection, and positive samples were shipped to RITM for molecular analysis in accordance with the guidelines for shipping Category B infectious substances [21].

### FAT

FATs were repeated at the RITM to confirm positive results. In brief, brain impression smears of the samples were fixed in cold acetone and then air-dried. Each smear was flooded with fluorescent isothiocyanate antirabies monoclonal globulin (Fujirebio Diagnostics, Inc., Malvern, PA, USA) in 1% Evan's blue counterstain and were incubated in a humid chamber at 37°C for 30 min. The slides were washed twice with phosphate-buffered saline, rinsed with double-distilled water (ddH_2_O), and air-dried. Buffered glycerol mounting medium was applied before viewing rabies virus antigens under an immunofluorescent microscope.

### PCR

Total RNA was extracted from animal brain tissues using a RNeasy Mini Kit (Qiagen, Hilden, Germany). Approximately 30 mg of tissue was homogenized using a homogenization pestle, and RNA was purified according to the manufacturer's protocol for animal tissues. RNA was eluted in 50 µL of diethylpyrocarbonate-treated water and stored at −70°C until further processing. The rabies virus G gene was amplified using primers RV-7F and RV-9RPh to generate a 2,425-bp amplicon (Table 1). Primers p1 and 304 were used to amplify the rabies virus N gene, thereby generating a 1,511-bp amplicon (Table 1).

**Table 1:** Primer list.

Primer	Target gene	Sequence (5′―3′)	Position*
RV-7F (Forward)	G	CTA TGG TCT GAC ATG TCT CTT CAG	3032–3055
RV-7R (Reverse)	G	CCC ATG TTC CAT CCA TAA GTC TAA G	4068–4092
RV-8F (Forward)	G	GAA GAT GGC CGG TGA CCC CAG ATA TG	3671–3696
RV-8R (Reverse)	G	CCA ACA ACT CCA TAT GTT GCY GGA GG	4511–4536
RV-9F (Forward)	G	GGG TTT GGA AAA GCA TAT ACC ATA TTC	4299–4325
RV-9RPh (Reverse)	G	TCA ACC GGG TCA TCA TAG ACC TCA CC	5421–5446
p1 (Forward)	N	ACA GAC AGC GTC AAT TGC AAA GC	28–50
304 (Reverse)	N	TTG ACG AAG ATC TTG CTC AT	1515–1534
N12 (Forward)	N	GTA ACA CCT CTA CAA TGG	57–74
N40 (Reverse)	N	GCT TGA TGA TTG GAA CTG A	1350–1368

Primers used in this study.

Amplification was performed using Superscript III One-Step RT-PCR with Platinum *Taq* High Fidelity Polymerase (Invitrogen, Carlsbad, CA, USA). The 25 µL RT-PCR reaction mixture contained 9 µL of ddH_2_O, 12.5 µL of 2× reaction mix, 0.25 µL of each primer at 50 µM, 0.5 µL of the enzyme mix, and 2.5 µL of RNA template. One step RT-PCR was performed using a TaKaRa PCR Thermal Cycler Dice (TaKaRa Bio Inc., Shiga, Japan) under the following thermocycling conditions for the G gene: reverse transcription at 50°C for 30 min, denaturation at 95°C for 5 min, 25 cycles of 94°C for 1 min, 65°C for 1 min, and 68°C for 1.5 min, and a final extension at 68°C for 10 min. The following thermocycling conditions were used for the N gene: reverse transcription at 50°C for 30 min, denaturation at 95°C for 5 min, 25 cycles of 94°C for 1 min, 54°C for 1 min, and 68°C for 1.5 min, and a final extension at 68°C for 10 min.

### Nucleotide Sequencing

A SUPREC PCR Purification Kit (TaKaRa Bio Inc.) or a QIAquick PCR Purification Kit (Qiagen) was used to purify RT-PCR products prior to nucleotide sequencing. Cycle sequencing was performed using a BigDye Terminator v1.1 or v3.1 Cycle Sequencing Kit (Applied BioSystems, Foster City, CA, USA) in the TaKaRa PCR Thermal Cycler Dice. Sequencing reactions were purified using a BigDye XTerminator Purification Kit (Applied BioSystems) followed by loading into Genetic DNA Analyzers 310, 3130, or 3730xl (Applied BioSystems). Bidirectional sequencing was performed using primers listed in Table 1 to resolve the complete G gene (1,572 nt) and a partial region (1,124 nt) of the N gene.

### Sequence Alignment and Genetic Analysis

Multiple sequence alignments and phylogenetic relationships were inferred using the maximum-likelihood method of the Molecular Evolutionary Genetics Analysis version 5 algorithm (MEGA5; http://www.megasoftware.net/) with bootstrap probability calculated from 500 replicates. In this analysis, the general time reversible+γ model was selected as a substitution model based on AICc values (Akaike Information Criterion, corrected) using the model selection feature of the MEGA5 program. The cut-off value for the condensed tree was set at 80% and was used to define genogroups. Clades and subclades were defined when the genogroup comprised more than 4 strains. Genogroups consisting of fewer than 3 strains were not considered as clades and are indicated by the prefix “Gr.” All reference sequences used for comparative analyses were obtained from the GenBank genetic sequence database (http://www.ncbi.nlm.nih.gov/genbank/; Table 2). Sequences described in this study were submitted to GenBank and corresponding accession numbers are listed in Figure S1.

**Table 2:** List of rabies virus samples obtained from other countries.

Sequence ID	Cluster	Country	Host	Year	Gene	Accession No.
RV/JSL26/2005	Asian 1a	China	Dog	2005	N	EU159381
					G	DQ849066
RV/JSL29/2005	Asian 1a	China	Dog	2005	N	EU159383
					G	DQ849068
RV/03003INDO/2003	Asian 1b	Indonesia	Dog	2003	N	EU086192
					G	EU086151
RV/CQ92/1992	Asian 1c	China	Dog	1992	N	EU159388
					G	DQ849072
RV/02050CHI/1992	Asian 1c	China	Human	1992	N	EU086185
					G	EU086145
RV/Guizhou_Al01/2005	Asian 2a1	China	Dog	2005	N	DQ666289
					G	EU267746
RV/GX4/1994	Asian 2a1	China	Dog	1994	N	EU159386
					G	DQ849071
RV/ZJ-LA/2008	Asian 2a2	China	Ferret badger	2008	N	FJ598135
					G	FJ719756
RV/JX08-48/2008	Asian 2a2	China	Ferret badger	2008	N	FJ719753
					G	FJ719749
RV/04030PHI/2004	Asian 2b	Philippines	Dog	2004	N	EU086205
					G	EU086155
RV/9913BIR/1999	Asian 2c	Myanmar	Dog	1999	N	EU086165
					G	EU086129
RV/01016VNM/2001	Asian 2c	Vietnam	Dog	2001	N	EU086209
					G	EU086159
RV/8734THA/1983	Asian 2c	Thailand	Human	1983	N	EU086206
					G	EU086157

The reference strains that were used from the Genbank to create Figure 2 have been listed. The cluster was determined by Gong et al (2009).

## Results

### Analyzed Samples

Of 317 brain tissue samples collected, 51 were excluded because of incomplete epidemiological information (5 samples), failure of PCR amplification (21 samples), or sequencing (25 samples). The complete G gene (1572 nt) and parital G gene sequences were determined for 233 and 33 strains, respectively.

**Figure 1:**
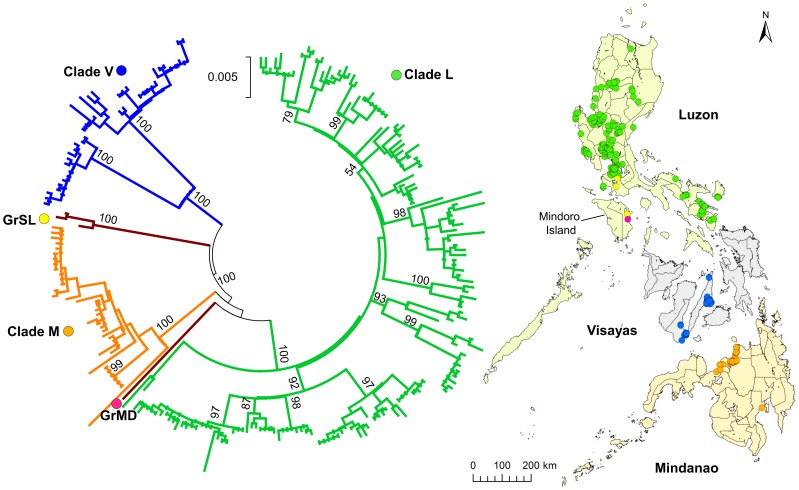
Phylogenetic tree of complete and partial G genes from 235 Philippine rabies virus strains. The phylogenetic tree of 233 complete (1572 nt) and two partial (1549 nt) G genes was constructed using the maximum-likelihood method and bootstrap values were calculated from 500 replicates. The three major clades, Luzon (green), Visayas (blue), Mindanao (orange), and two distinct genogroups, Mindoro (pink), and South Luzon (yellow), are indicated with different colors. Dots on the map indicate the number and collection sites of each sample (one dot = one sample). Bootstrap values of greater than 90% are presented.

A phylogenetic tree of partial G genes (698 nt) from 266 strains was obtained (Figure S2), and because of low bootstrap values, only samples with complete G genes were included in the analysis. However, as a minor but unique branch was found in the phylogenetic tree of partial G genes (698 nt), two samples collected from Batangas and Mindoro Island were included despite incomplete sequences (1549 nt; Figure S2). Therefore a total of 235 samples were analyzed (Figure 1).

Of the 235 samples, 35 were collected from 2004 to 2007 and deposited in the sample bank, while 200 were collected prospectively from 2008 to 2010. Among analyzed samples, 228 were collected from dogs, two from cats, one from a cow, and four from unknown host species (Tables 3 and S1).

**Table 3:** Classification of analyzed samples according to region and year of collection.

Island group	Luzon								Visayas	Mindanao		
Region	1	2	CAR	3	NCR	4A	4B	5	7	10	11	Total
**2004**	0	0	0	1	2	4	0	0	0	0	0	**7**
**2005**	0	0	0	2	8	0	0	0	0	0	1	**11**
**2006**	0	0	0	0	0	11	2	0	0	0	0	**13**
**2007**	0	0	0	0	0	4	0	0	0	0	0	**4**
**2008**	1	0	0	39	0	0	0	13	17	16	0	**86**
**2009**	27	4	7	20	0	0	0	9	23	20	0	**110**
**2010**	3	0	1	0	0	0	0	0	0	0	0	**4**
**Total**	**31**	**4**	**8**	**62**	**10**	**19**	**2**	**22**	**40**	**36**	**1**	**235**

The regional and annual distributions of the 235 samples collected from the Philippines are listed. Samples collected in 2004–2007 were retrieved from the sample bank of RITM. The samples of 2008–2010 were prospectively collected from RADDLs and PADDL.

### Phylogenetic Analysis

**Figure 2:**
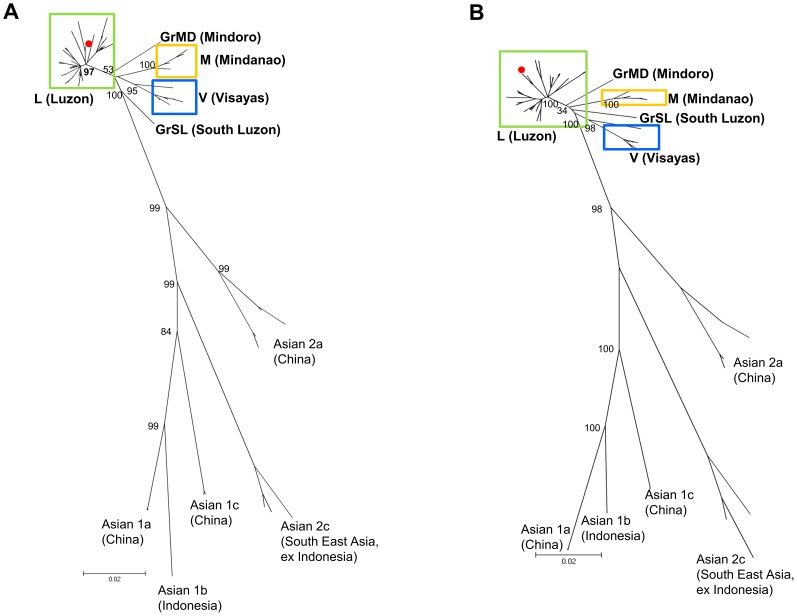
Comparison between phylogenetic trees of rabies virus N and G genes. Comparison of rabies virus phylogenetic trees for the (A) partial N gene (1124 nt) and the (B) complete G gene (1572 nt) using 57 virus strains from the Philippines and other Asian countries. The trees were constructed using the maximum-likelihood method and bootstrap values were calculated from 500 replicates. Both trees show the three major clades, Luzon (green line), Visayas (blue line), and Mindanao (orange line), and the two distinct strains from Mindoro and South Luzon. The red circle shows the Philippine strain (RV/04030PHI/2004) [16], [19] from the cluster that was previously named Asian 2b by Gong et al [19].

From 235 samples analyzed, phylogenetic trees were constructed using 57 strains of N (1124 nt) and G (1572 nt) genes with the maximum-likelihood method. To determine phylogenetic relationships, phylogenetic trees for the N and G genes were constructed to compare the Philippine strains with those from other Asian countries (Figure 2A and 2B). All the Philippine strains were classified into one distinct cluster, which was divergent from those of other Asian countries. This cluster included only the Philippine strains and was previously named Asian 2b by Gong et al. [22]. In the phylogenetic tree, the cluster closest to the Philippine strains was Asian 2a, which comprised strains from China, as shown in previous studies [16], [22], [23]. The Philippine cluster was further divided into three major clades, namely L (Luzon), V (Visayas), and M (Mindanao). However, two strains from southern Luzon and Mindoro Island, namely GrSL and GrMD, respectively, were not classified into any of the 3 major clades in either N or G phylogenetic trees (Figure 2A and 2B).

To investigate phylogenetic relationships between the Philippine strains in detail, we analyzed the full-length G gene sequence from 235 strains and the partial G gene (1549 nt) from two strains. It was confirmed that there were three major clades and two distinct genogroups. Clade L and two genogroups were found in the island group of Luzon, whereas clades V and M were found in the specific geographic island groups Visayas and Mindanao, respectively. Interestingly, strains of the distinct genogroups GrSL and GrMD were found in Mindoro Island and none of the clade L strains were found on this island (Figure 1).

**Figure 3:**
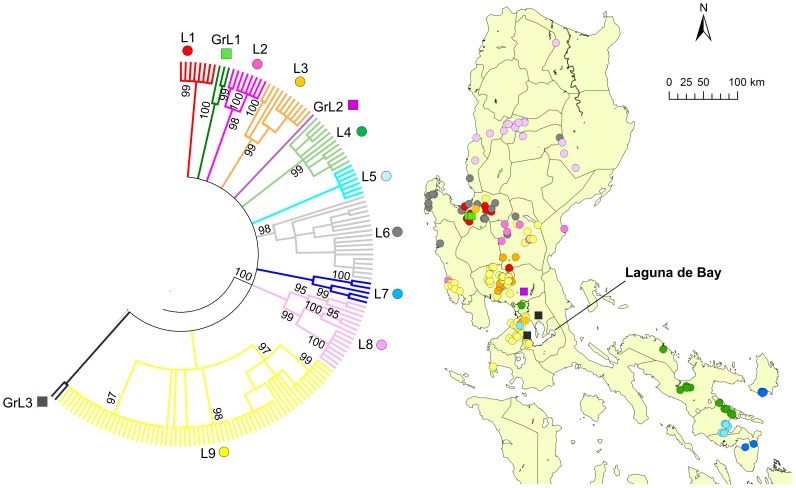
Geographic distribution of clade L. The phylogenetic tree for the G gene from 235 Philippine strains was constructed using the maximum-likelihood method and bootstrap values were calculated from 500 replicates. The condensed tree with the cut off value of 80% is presented and clade L is enlarged. There were nine subclades and three genogroups in clade L.

**Figure 4:**
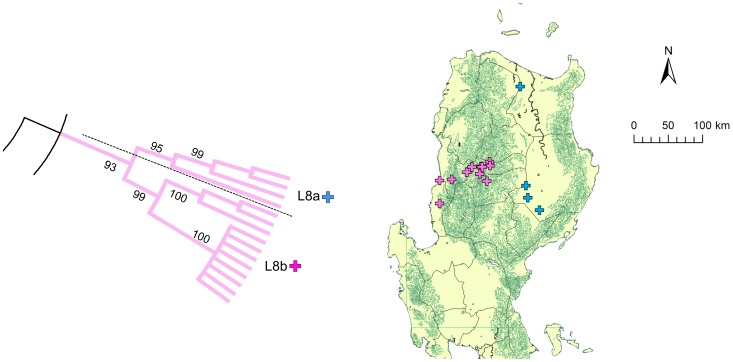
Geographic distribution of subclade L8. Subclade L8 in the phylogenetic tree of G genes from 235 Philippine strains is enlarged. L8 was further divided into two subclades, L8a and L8b. The topographical imagery in Luzon Island is shown with a green line. A condensed tree with a cut off value of 80% is presented.

**Figure 5:**
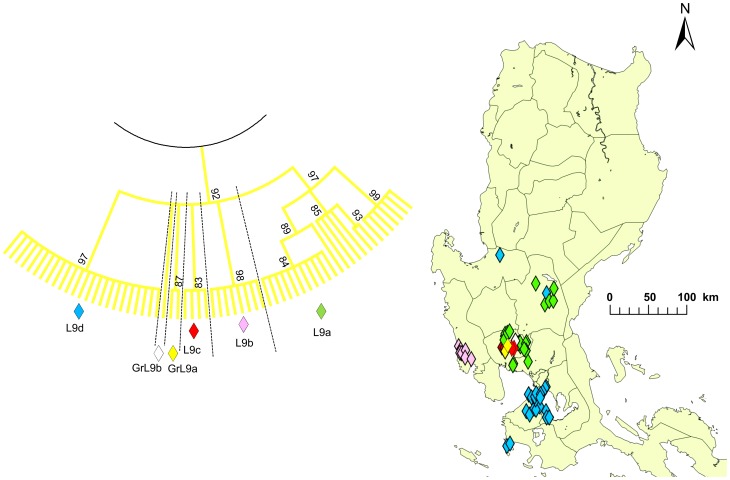
Geographic distribution of subclade L9. Subclade L9 in the phylogenetic tree of the G gene from 235 Philippine strains is enlarged. L9 was further divided into 4 subclades and 2 genogroups, L9a–L9d and GrL9a–GrL9b, respectively. A condensed tree with a cut off value of 80% is presented.

Clade L was further divided into nine subclades, L1–L9, and there were three distinct genogroups with less than four strains, which were named GrL1–GrL3 (Figure 3). L1 and GrL1 were found in the Pangasinan Province of the western part of central Luzon where L6 strains were also common, whereas L2 was found mainly in the eastern part of central Luzon (Figure 3) and L3 was also mainly distributed in central Luzon. GrL2 consisted of only one strain that was found in Pampanga Province, and GrL3 consisted of two strains that were distributed in the provinces of Rizal and Cavite located around Laguna de Bay (Figure 3). L4, L5, and L7 were found in southwestern Luzon (Region 5) with the exception of one strain each from L4 and L5, which were located in NCR (Figure 3). L8 was further divided into L8a and L8b (Figure 4). L8a and L8b strains were found in the northeastern and northwestern parts of Luzon Island, respectively. L9 was divided into 4 subclades, L9a–L9d, and 2 genogroups, GrL9a–GrL9b (Figure 5). L9a, L9c, GrL9a, and GrL9b were found in central Luzon, especially in Pampanga Province, and L9b was found exclusively in Zambales Province. L9d was distributed in NCR and Region 4A, with the exception of two strains located in the provinces of Nueva Ecija and Pangasinan.

**Figure 6:**
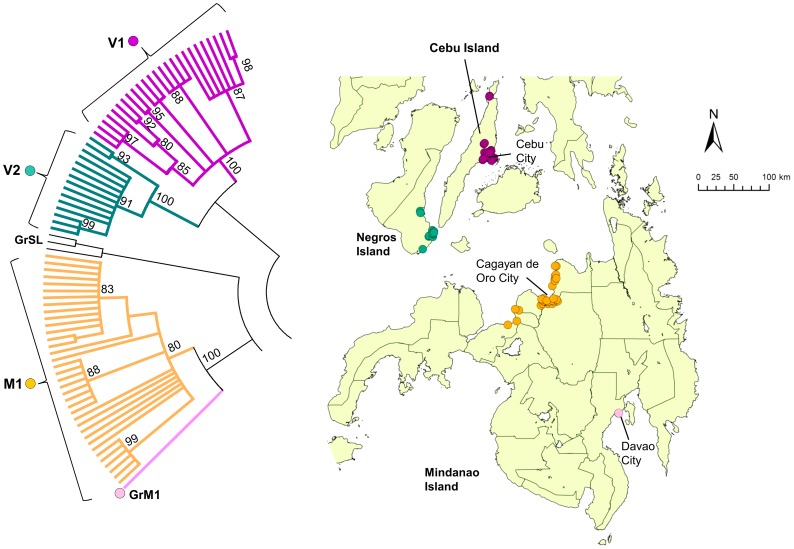
Geographic distribution of clade V and M. Clades V and M in the phylogenetic tree of the G gene from 235 Philippine strains is enlarged. Clade V was subdivided into two subclades, V1 and V2, and clade M comprised one subclade (M1) and 1 genogroup (GrM1). Bootstrap value of more than 80% was observed.

Clade V (Visayas) was subdivided into the two subclades V1 and V2. All V1 strains were found exclusively on Cebu Island, whereas V2 strains were found exclusively on Negros Island (Figure 6). Clade M (Mindanao) strains consisted of only one subclade (M1) and one genogroup (GrM1), and all samples except 1 was from northern Mindanao. The single sample from Davao (GrM1; southern Mindanao) was distinct from all other strains from this island (Figure 6).

## Discussion

The N and G genes of rabies field strains obtained from 11 of 17 regions in the Philippines were analyzed. Our phylogenetic analysis of the N and G genes revealed that there were three major clades, namely L, V, and M (Figure 2). A previous study identified two distinct clades in the Philippines [9]; however, it was based on the analysis of only 59 strains and detailed geographic information was not provided. In this study, we analyzed more systematically collected strains from 11 different regions, including Luzon, Visayas, and Mindanao, and identified a previously unknown clade, clade V, from Visayas. The presence of two major clades from Luzon, Visayas, and Mindanao indicated that rabies viruses have evolved independently in these island groups mainly because they are physically separated by sea. Similar geographic compartmentalization of rabies viruses has been reported in Indonesia, which is also an island nation [11].

Four strains were not classified into any of the three major clades, including three in GrSL and another in GrMD (Figure 1). Because only a few samples were found containing these genogroups, evolutionary relationships with major clades could not be defined. GrSL was found both in Luzon and Mindoro Islands, which indicates that viral transmission occurred between these islands. A similar pattern was observed in L7, which was found in Sorsogon in Luzon Island and Catanduanes Island. This suggests that even in areas separated by the sea, such as a narrow channel with frequent ferry traffic, complete geographic barriers for rabies transmission may not be established and that inter-island transmission is possible in some exceptional cases. It is possible that GrSL and GrMD formed distinct major clades, though this remains to be confirmed with analyses of more strains in these genogroups. There was also unique genogroup in clade L, i.e. GrL3 was diverted separately from other subclades in clade L with a bootstrap value of 42% (data not shown). Interestingly, all the strains in these genogroups (GrMD, GrSL, and GrL3) were found in southern Luzon and its neighboring island Mindoro. More samples from these areas should be analyzed to better define evolutionary relationships.

A recent molecular evolutionary analysis indicated that rabies viruses in the Philippines (Asian 2b) diverged from viruses in China approximately 1813 (from 1707 to 1821) [22]. Another molecular evolutionary analysis based on N gene sequences suggested that the time of their divergence may have ranged from 1825 to 1936 [23]. It has been indicated that rabies viruses were introduced into previously rabies-free regions by human-mediated animal movements [24]. The introduction of rabies viruses into the Philippines was also possibly associated with human migration from China to the Philippines. Further molecular evolutionary analyses of rabies viruses in the Philippines are required to determine precise evolutionary histories.

In general, subclades of clade L were represented by viruses found in geographically distinct areas. Interestingly, L8a and L8b were clearly divided by a mountainous area (Mountain Province and Ifugao Province; Figure 4), indicating that the mountain range also had an important impact on rabies virus transmission. L9 was divided into four clades and two genogroups, indicating circulation within a small geographic area (Figure 5). In clade L9, only L9d was found in NCR and Region 4A from 2004 to 2007, whereas other genogroups were found in Region 3 during 2008 and 2009 (Figure S3). Thus, a possibility that subclustering is determined not only by geographical elements but also temporal elements cannot be ruled out and samples collected from NCR after 2008 are required to decipher these effects. The provinces of Pampanga and Nueva Ecija contained five subclades and branches each, and the Pangasinan Provinces and NCR contained four branches each (Figures 3 and 5). Careful monitoring of the circulating strains between these areas, in which multiple subclades were found may provide important information regarding temporal and geographic transmission patterns of viruses in the Philippines.

In Region 5 of Luzon, the three subclades L4, L5, and L7 (Figure 3) were present. All L4 and L5 strains were found in Region 5, with the exception of one strain of each found in NCR (Figure 3). These long distance disseminations of viruses may have occurred through human-mediated dog movements [11]. However, it is unclear with the current data whether these originated in NCR or Region 5. Similarly, all of the L6 strains were distributed in Regions 3 and 5, with the exception of one strain found over the mountain range in the CAR.

In Visayas, viruses from different subclades V1 and V2 exist on the Cebu and Negros islands, respectively (Figure 6). Although these are separated by a narrow strait, our molecular analysis indicated that there was no viral movement between the two islands. All of the samples from Mindanao Island, with the exception of a sample from Davao, were collected from the northern region, particularly around the city of Cagayan de Oro (Figure 6). More samples from other areas are required to elucidate subclades from Mindanao Island. Like other islands, Mindanao may have been the site of a unique viral evolution.

The Philippines has a national goal of eliminating rabies by the year 2020, and a campaign of dog vaccinations has been conducted by each local government. However, the program has not been systematically implemented and funding allocation depends on the priorities of each local government. Hence, there is an urgent need to develop a more effective and feasible strategy to control rabies. Being an island nation, the Philippines has an advantage as rabies has been successfully eliminated in other island nations such as the UK and Japan [25], [26]. Our molecular analyses showed that most rabies virus strains in the Philippines are uniquely clustered, suggesting that introduction from other countries has not occurred recently.

Understanding the transmission dynamics of the rabies viruses is necessary for the development of an effective and feasible elimination strategy, particularly in countries with limited resources. Because of financial and resource constraints, the NRPCP of the Philippines adopted a phasing approach to eliminate rabies by area rather than nation-wide implementation of extensive dog vaccinations. The effectiveness of this strategy relies on limited transmissions between areas. Our molecular analyses indicated that this strategy could be effective in controlling rabies in the Philippines because there was clear geographic clustering of clades and subclades. However, a synchronized campaign would be necessary if there is frequent inter-island traffic. The Republic Act No. 9482, known as the Anti-Rabies Act of 2007, was enacted to control and eliminate human and animal rabies. This mandated control over dog and cat movements during inter-island transport and involved valid vaccination certifications only allowing transport two weeks or more after vaccination or within 12 months of vaccination by a licensed veterinarian [27]. This policy must be strictly implemented to avoid inter-island virus transmission via animal transportation as observed for the GrSL strain between Luzon and Mindoro Islands and the L7 strain between Luzon and Catanduanes Islands.

In some areas of Thailand, homogeneous viruses circulated without recent introductions, which can serve as initial control targets [13]. However, the introduction of new genotypes to other locations is likely because of human-mediated dog movements [13]. We found several instances where viral dispersal may have occurred with dog movements. However, the frequency of such dispersal appeared to be much lower than that in Thailand. A more detailed analysis of dog movements, particularly on Luzon Island, which is the largest island in the Philippines, should be conducted to evaluate the risk of geographic dispersal.

There were several limitations to our study. Firstly, we analyzed strains from wide geographic areas, including 11 of the 17 regions of the Philippines; however, many areas remain unexamined. The number of samples analyzed per region also varied because some regional laboratories tested samples more actively than others. Further, more samples were tested in areas close to the regional laboratory. Therefore, samples from more remote areas were not included in the analysis. Lastly, due to the limited time interval of sample collection, we only analyzed geographic clustering of rabies virus in the Philippines and temporal analysis, including molecular evolutional analyses were not conducted in this study. Further studies are required to fully understand these viral evolutionary processes. Despite these limitations, our analysis provides valuable molecular phylogeny and spatial distribution data for rabies virus variants currently circulating in the Philippines.

## Supporting Information

Figure S1 **Sites of sample collection.** Sampling sites are indicted with red edges and large font labels. Regions with color and small font labels indicate sites of sample submission. The color code indicates the three major island groups Luzon (green), Visayas (blue), and Mindanao (orange).
(TIF)

Figure S2 **Phylogenetic tree of the partial G gene (698 nt) from 266 Philippine strains.** The phylogenetic tree of the partial G gene (698 nt) from 266 Philippine strains was constructed using the maximum-likelihood method and bootstrap values were calculated from 500 replicates. Three major clades, Luzon (green), Visayas (blue), Mindanao (orange), and two distinct genogroups, Mindoro (pink), and South Luzon (yellow), are indicated with different colors. Samples determined only with partial G gene sequences are indicated in red or green. Bootstrap values of more than 90% are presented. Strains with incomplete sequence data were excluded from the main analysis (red circle), except for the samples belonging to GrSL (green square).
(TIF)

Figure S3 **Phylogenetic tree with collection year.** The entire phylogenetic tree of the G gene from 235 Philippine strains was constructed using the maximum-likelihood model. Circles are color coded according to the year of collection of each sample. The names of branches or clades determined in this study are indicated.
(TIF)

Table S1 **Rabies virus strains obtained during this study.** The names of subclades or genogroups and their accession numbers are listed for each of the rabies virus strains. Subclades and genogroups were determined using complete or partial G gene sequence data.
(XLSX)
