# Correction: Genetic Diversity and Geographic Distribution of Genetically Distinct Rabies Viruses in the Philippines

**DOI:** 10.1371/annotation/cf5aae8c-03f4-49d5-94e8-06b4f73847f9

**Published:** 2013-07-30

**Authors:** Mariko Saito, Hitoshi Oshitani, Jun Ryan C. Orbina, Kentaro Tohma, Alice S. de Guzman, Taro Kamigaki, Catalino S. Demetria, Daria L. Manalo, Mary Elizabeth G. Miranda, Akira Noguchi, Satoshi Inoue, Beatriz P. Quiambao

Mary Elizabeth G. Miranda was not included in the author byline. She should be listed as the ninth author and her institutional affiliation is the Research Institute for Tropical Medicine, Muntinlupa City, Metro Manila, Philippines. Her contributions include: conceived and designed the experiments.

In addition, there are errors and incomplete data in Table S1. Please view the correct Table S1 here: http://www.plosntds.org/article/info%3Adoi%2F10.1371%2Fjournal.pntd.0002144

