# Supplementary figures and images for: Genetic Diversity and Geographic Distribution of Genetically Distinct Rabies Viruses in the Philippines
Source: PLoS Negl Trop Dis. 2013 Apr 4;7(4):e2144. doi: 10.1371/journal.pntd.0002144 (PMC3617229; doi:10.1371/journal.pntd.0002144)

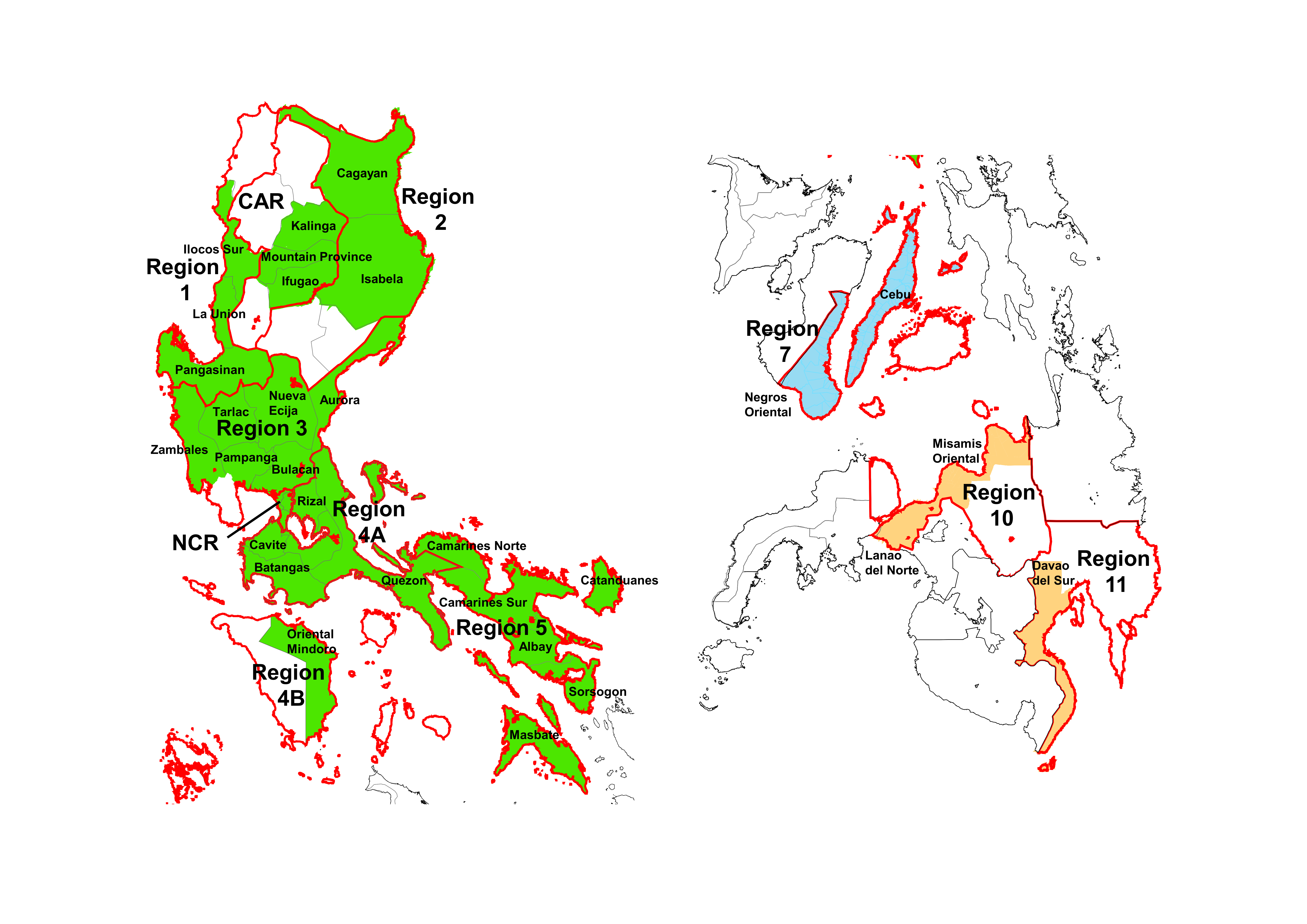

Supplement: Figure S1 — Sites of sample collection. Sampling sites are indicted with red edges and large font labels. Regions with color and small font labels indicate sites of sample submission. The color code indicates the three major island groups Luzon (green), Visayas (blue), and Mindanao (orange). (TIF) [file pntd.0002144.s001.tif]

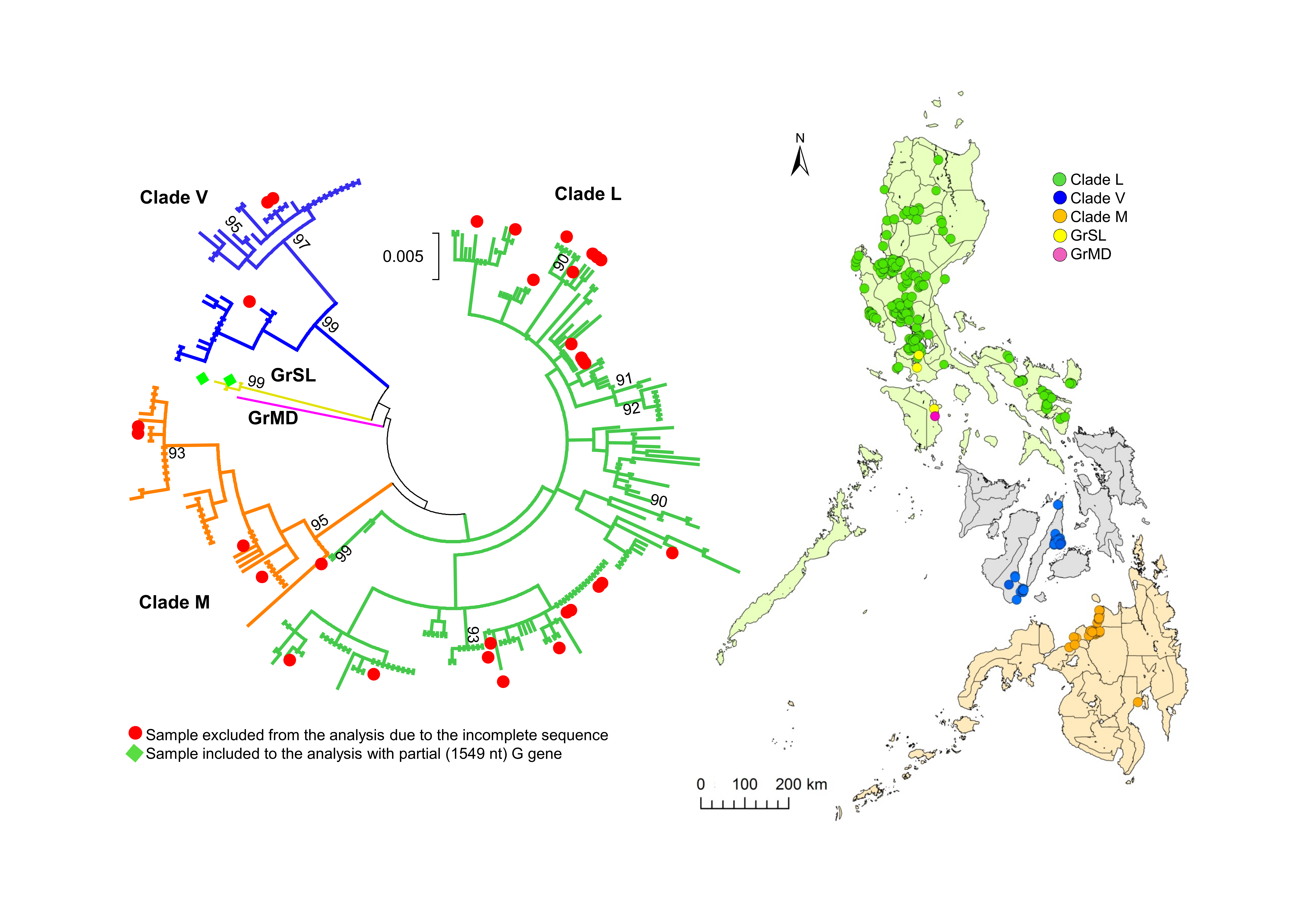

Supplement: Figure S2 — Phylogenetic tree of the partial G gene (698 nt) from 266 Philippine strains. The phylogenetic tree of the partial G gene (698 nt) from 266 Philippine strains was constructed using the maximum-likelihood method and bootstrap values were calculated from 500 replicates. Three major clades, Luzon (green), Visayas (blue), Mindanao (orange), and two distinct genogroups, Mindoro (pink), and South Luzon (yellow), are indicated with different colors. Samples determined only with partial G gene sequences are indicated in red or green. Bootstrap values of more than 90% are presented. Strains with incomplete sequence data were excluded from the main analysis (red circle), except for the samples belonging to GrSL (green square). (TIF) [file pntd.0002144.s002.tif]

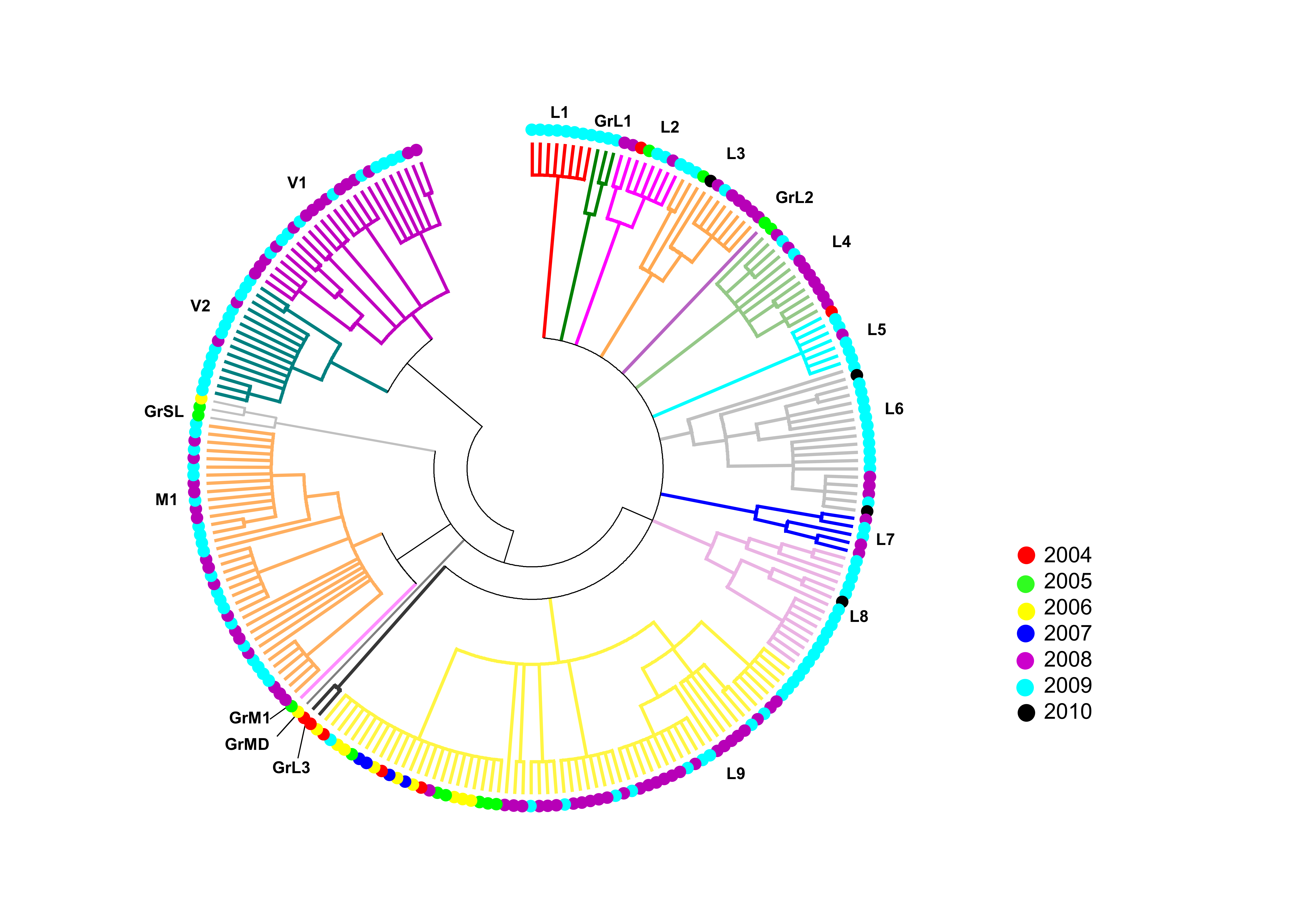

Supplement: Figure S3 — Phylogenetic tree with collection year. The entire phylogenetic tree of the G gene from 235 Philippine strains was constructed using the maximum-likelihood model. Circles are color coded according to the year of collection of each sample. The names of branches or clades determined in this study are indicated. (TIF) [file pntd.0002144.s003.tif]
